# Integrative workflows for metagenomic analysis

**DOI:** 10.3389/fcell.2014.00070

**Published:** 2014-11-19

**Authors:** Efthymios Ladoukakis, Fragiskos N. Kolisis, Aristotelis A. Chatziioannou

**Affiliations:** ^1^Laboratory of Biotechnology, Department of Chemical Engineering, School of Chemical Engineering, National Technical University of AthensAthens, Greece; ^2^Metabolic Engineering and Bioinformatics Program, Institute of Biology, Medicinal Chemistry and Biotechnology, National Hellenic Research FoundationAthens, Greece

**Keywords:** metagenomics, bioinformatics, distributed computing, cloud computing, workflow engines

## Abstract

The rapid evolution of all sequencing technologies, described by the term Next Generation Sequencing (NGS), have revolutionized metagenomic analysis. They constitute a combination of high-throughput analytical protocols, coupled to delicate measuring techniques, in order to potentially discover, properly assemble and map allelic sequences to the correct genomes, achieving particularly high yields for only a fraction of the cost of traditional processes (i.e., Sanger). From a bioinformatic perspective, this boils down to many GB of data being generated from each single sequencing experiment, rendering the management or even the storage, critical bottlenecks with respect to the overall analytical endeavor. The enormous complexity is even more aggravated by the versatility of the processing steps available, represented by the numerous bioinformatic tools that are essential, for each analytical task, in order to fully unveil the genetic content of a metagenomic dataset. These disparate tasks range from simple, nonetheless non-trivial, quality control of raw data to exceptionally complex protein annotation procedures, requesting a high level of expertise for their proper application or the neat implementation of the whole workflow. Furthermore, a bioinformatic analysis of such scale, requires grand computational resources, imposing as the sole realistic solution, the utilization of cloud computing infrastructures. In this review article we discuss different, integrative, bioinformatic solutions available, which address the aforementioned issues, by performing a critical assessment of the available automated pipelines for data management, quality control, and annotation of metagenomic data, embracing various, major sequencing technologies and applications.

## Introduction

**Figure 1:**
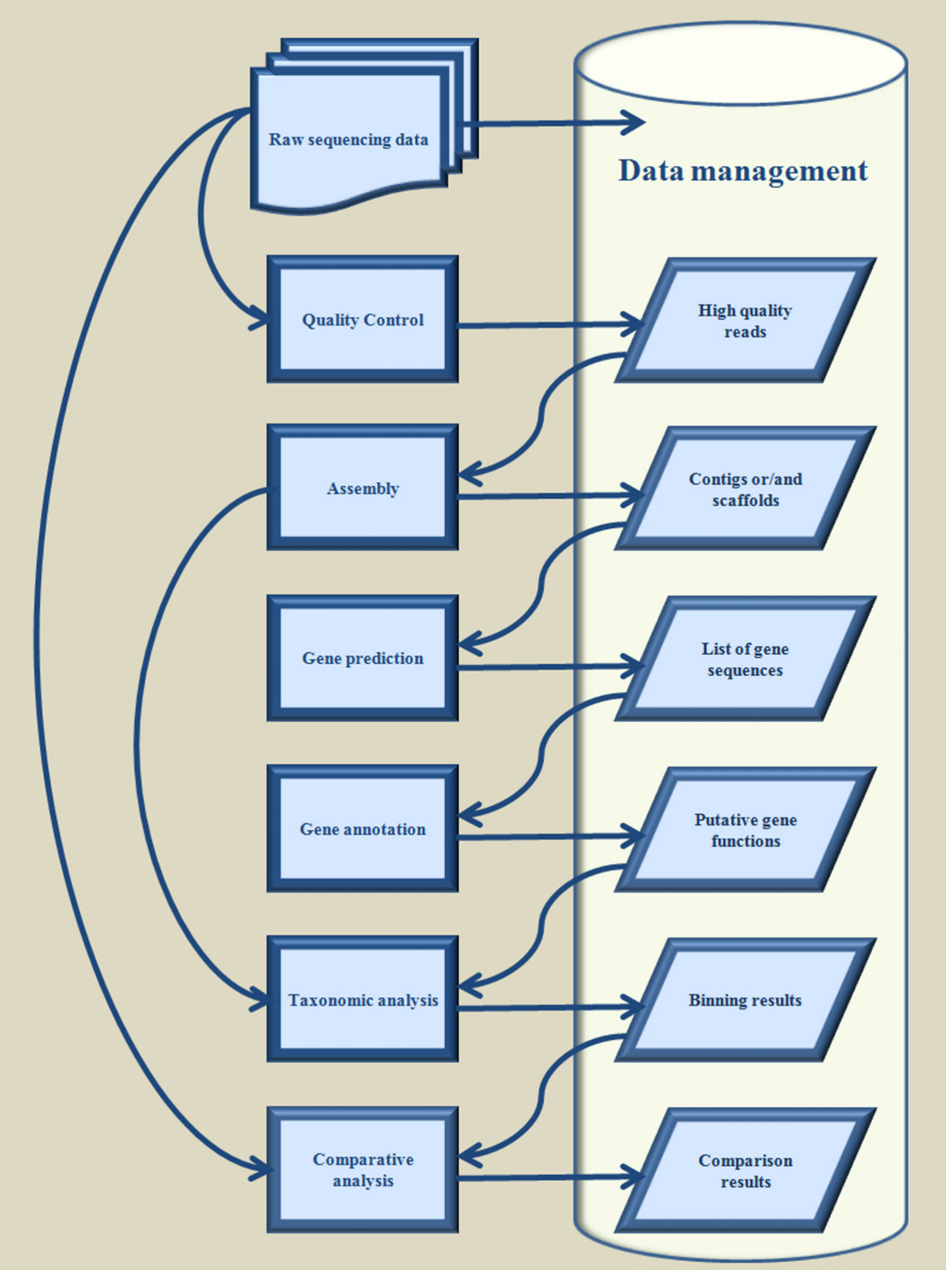
**Typical workflow for analysis of metagenomic sequencing data**.

Metagenomics refers to the exhaustive study of a collection of genetic material, encompassing various genomes from a mixed community of organisms as defined from the National Human Genome Research Institute (*Talking Glossary of Genetic Terms^1^*). The definition embraces the cases where either the sampling is conducted, in an environmental habitat, or the material is collected from the tissue of a particular host organism, aiming to unravel the complexity of the microbial species, which are adapted to cooperate through symbiotic modes. The scrupulous study of a metagenome (Handelsman et al., 1998) offers insight concerning not only the phylogenetic properties of the environmental niche itself, but also of its exceptionally abundant arsenal of enzymes while, at the same time, provides us with a “recipe” to recreate or even redesign them *in vitro*, for the sake of various biotechnological applications. Genomic information acquired from metagenomic sampling, has become a fundamental step for the elucidation of the taxonomic composition of the niche together with each organism's potent enzymatic capabilities and is derived through the proper analysis of the chunks of DNA sequences, i.e., the full documentation of the nucleotide sequences that constitute the metagenome that are generated from a metagenomic sequencing experiment. Sequencing techniques have greatly evolved (Metzker, 2010b) the last decade and exploiting a variety of high-throughput protocols, so as to achieve exceptionally high yields for only a fraction of the cost of traditional processes (i.e., Sanger sequencing, Sanger et al., 1977). This evolution has resulted in a massive outbreak of data that are becoming increasingly hard to process due to their size and the numerous different tools essential for each step of the analytical endeavor. A thorough analysis of a metagenomic sample requests certain successive bioinformatic tasks that comprise (i) quality control, (ii) assembly, (iii) gene detection, (iv) gene annotation, (v) taxonomic analysis, and (vi) comparative analysis, whilst storing the generated results under a database-structured computational repository enabling advanced data management, processing, mining, and meta-mining capabilities (Figure 1). Each stage in this succession of bioinformatic tasks necessitates substantive expertise concerning the apposite utilization of the given software tool or algorithm, something that concerns either the mathematical concepts underlying the operation of a tool, or knowledge about programming aspects of its implementation and performance. The complexity of these tasks augments radically, with an increasing number of analyses. Recently, many bioinformatic pipelines have emerged that aim to address these issues through the provision of automated workflows and user friendly interfaces, in an effort to simplify the analytical procedure as much as possible, and minimize the entry barrier concerning the familiarization of the user with advanced programming or computational techniques. Each of these integrative analysis pipelines encapsulates a plethora of bioinformatic algorithms, seamlessly embedded into a multi-tasking framework that can address all aspects of a complete metagenomic analysis in an automated fashion. In this review we perform an appraisal of the available solutions of this kind for metagenomic purposes, by describing their configuration and their particular operational features, together with an assessment of their pros and cons, while we propose the most appropriate ones for particular analytical tasks.

## Data acquisition

**Figure 2:**
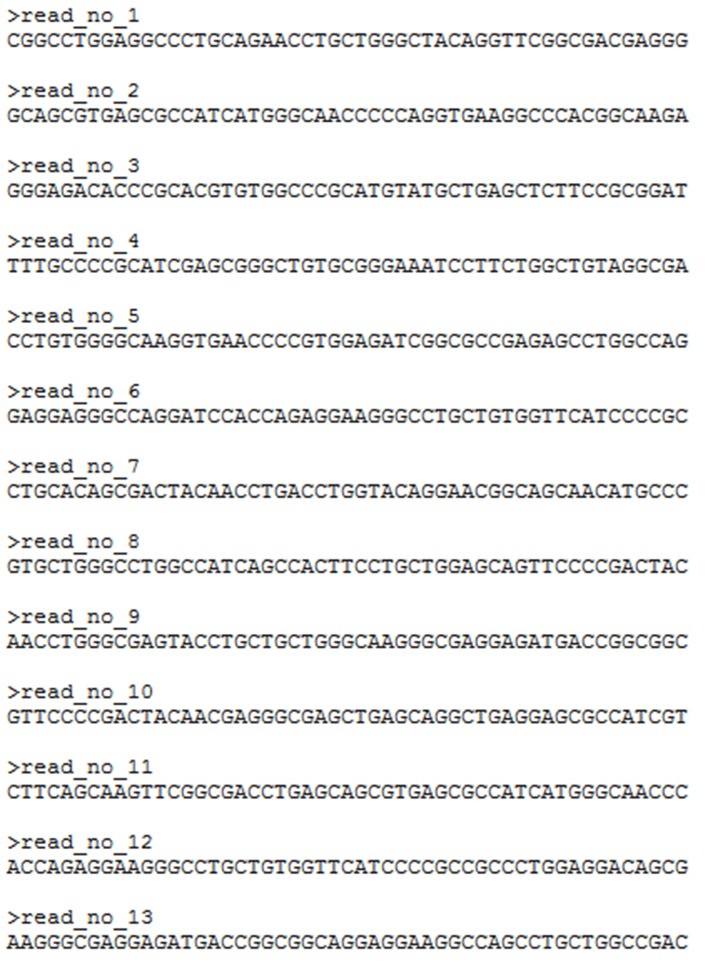
**Raw sequence reads in FASTA format**.

**Figure 3:**
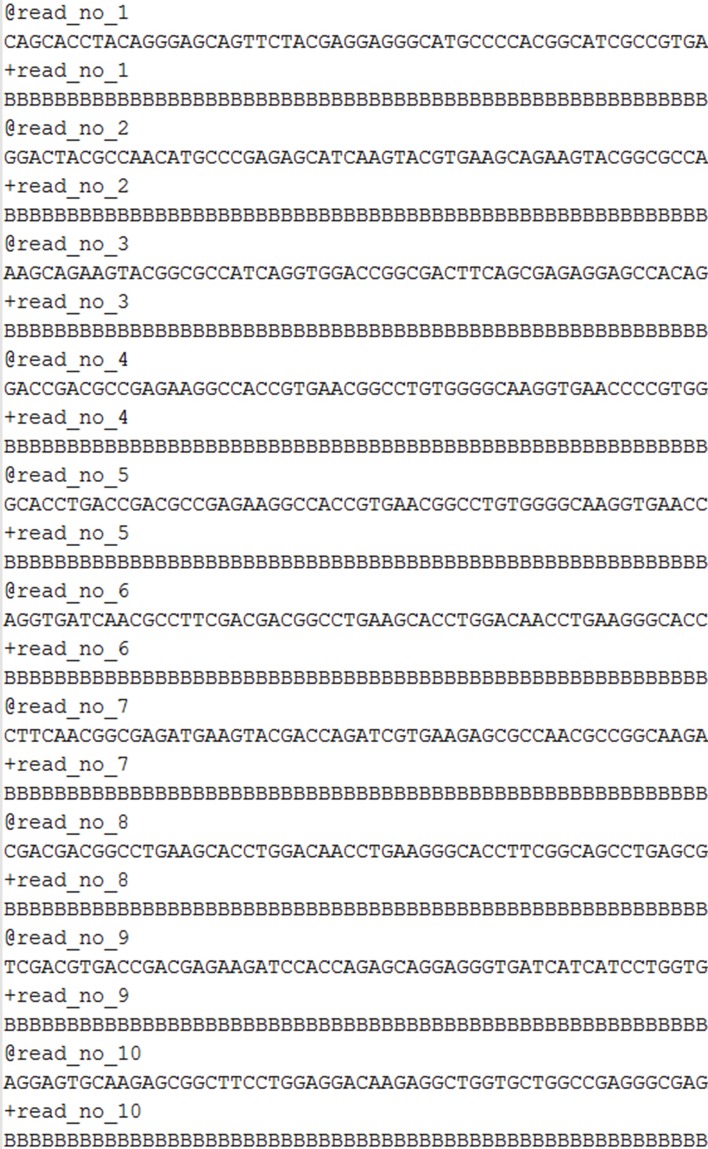
**Raw sequence reads in FASTQ format**.

There are numerous protocols available for environmental sample collection, metagenomic DNA extraction and amplification with several commercial kits available on the market. The sequencing of the acquired metagenomic DNA either with traditional sequencing techniques (Sanger sequencing) or with Next Generation Sequencing (NGS) (Metzker, 2010a) methodologies provides data in the form of small nucleotide sequences (reads) that correspond to different amplified strands of the same DNA molecule(s) each of which is randomly sheared into smaller pieces (shotgun sequencing). The generated datasets consist of text files in FASTA (Figure 2) or FASTQ (Figure 3) format containing, in the case of a typical experiment, millions of such reads, which are used for the assembly (partial or complete) of the DNA strand from which they originated. These datasets correspond to data files, which size can level, according to the depth of the sequencing analysis and quality of the instrumentation, up to several GB, thus rendering their proper processing, an elaborate, intensive task.

### Development of analytical workflows

Despite the fact that the experimental implementation of a NGS experiment comprises a painstaking and arduous procedure, its output, namely the volumes of short sequence reads, in digitized format, represents just the initial step, for the whole analytical process, setting a point where the plethora of available data are totally illegible and non-comprehensible. In order to dig out the information hidden in these datasets, one needs to define elaborate, multi-step, bioinformatic analytical workflows that can be performed either serially or in parallel with each other. As such processing tasks are so profoundly versatile and complicated in their logical structure and programmatic development, that even an experienced team of programmers can only develop a handful of them. In this respect, the intricate nature of the various processing steps that need to be assembled together, in order to form computational workflows appropriate for different analytical tasks, strongly supports the formation of federated computational infrastructures, representing repositories of software services, that can be transparently, (namely without any knowledge about their internal architecture), integrated in the available workflows, or can compile new ones. The vision for the creation of a suitable collaborative, environment, for a long list of genomic sequence analysis tasks, representing an analog of a virtual laboratory, relies on the extent of automation, easiness in integration, transparency, and functional versatility it provides. Beneath, follows a rough account of the main processing modules, incorporated in the workflows developed for metagenomic analysis.

#### Quality control

**Figure 4:**
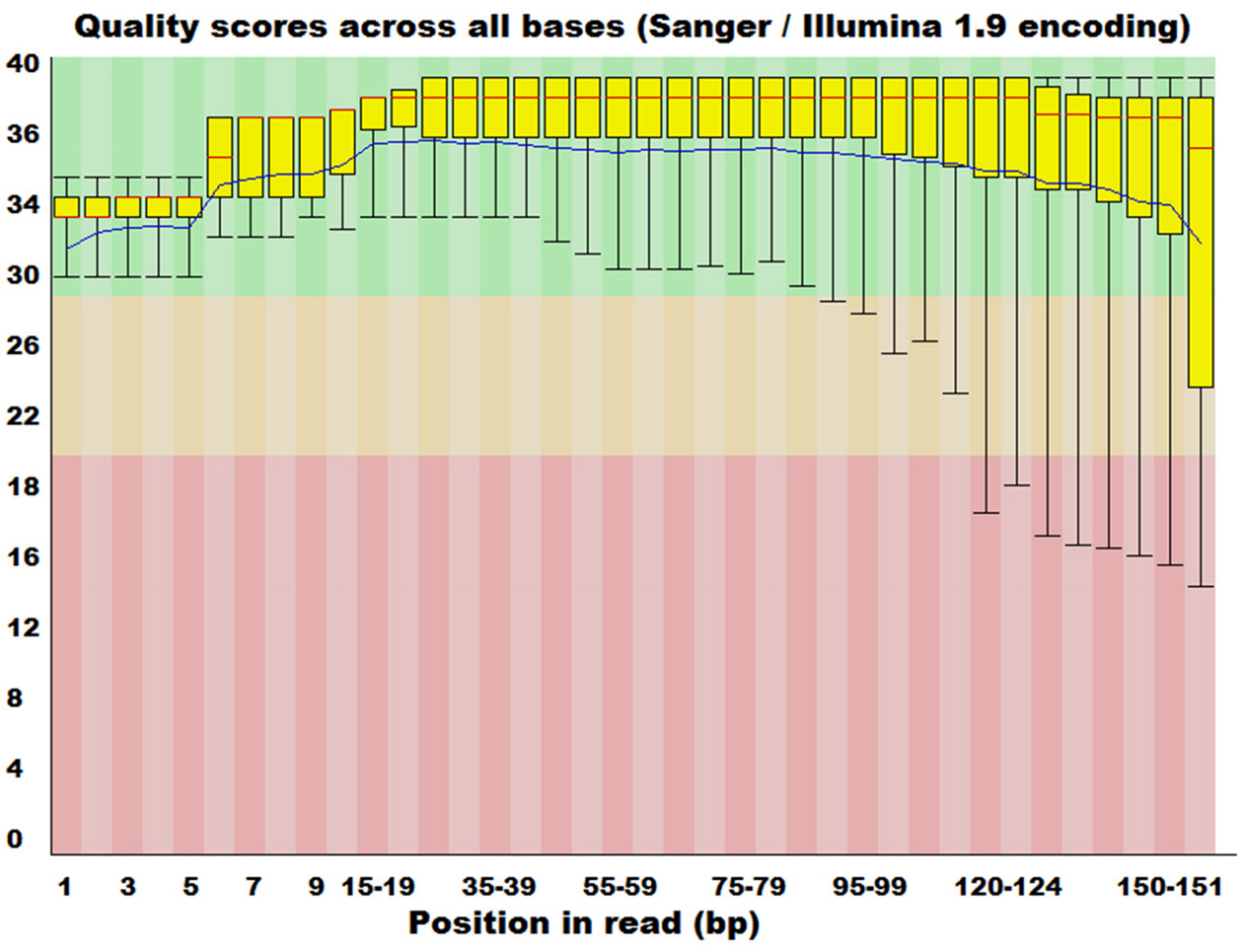
**Distribution of quality scores of raw sequence reads from FASTQC software**. Taxonomic sorting of sequencing reads from MEGAN software (rank level: “species”).

The genomic (DNA) material, isolated from a metagenomic sample, is transformed through the complicated experimental DNA sequencing protocols into short sequence reads of variable length, according to the protocols and instrumentation applied (Mardis, 2008; Shendure and Ji, 2008). This base calling procedure, is susceptible to bias depending on a number of factors (Clark and Whittam, 1992) such as G+C content and the actual location of the base in the sequence. This bias is quantified by measuring the probability of a base call to be false, providing an index of overall quality of the sequencing task. The computation of a quality score (Phred) (Cox et al., 2010; Schmieder and Edwards, 2011) for each sequenced base is now possible with this type of information being handily accommodated in the FASTQ file format, which represents a highly popular solution for genomic sequencing data exchange and storage, bearing both sequence and corresponding quality information (Cock et al., 2010). Several tools (Patel and Jain, 2012; Davis et al., 2013; Yang et al., 2013) have been developed that can utilize these scores and provide error probability distributions (Figure 4) as well as utilize appropriate filtering algorithms to trim sequences in a way that maintains only high quality genomic sequences.

#### Assembly

The next data processing step is the utilization of reads to assemble larger coherent sequence constructs (contigs) and, if possible, constructs that contain multiple contigs (scaffolds) with reliable connections between them. Each of these constructs originates from a different DNA sequence, that can be part of or a genome by itself and can be later investigated for the detection of open reading frames (ORFs), that is genomic areas, containing gene encoding sequences. The assembly task is so far, from the aspect of computational load, the bottleneck for any sequencing project whether the data corresponds to single cell genomes or metagenomic samples. The assembly of reads to contigs (and scaffolds) is a very laborious task, requesting avidly memory processing power resources, setting an important challenge, for which numerous algorithms (Miller et al., 2010) have been developed to address various performance issues stemming from it. Whereas there are numerous algorithms (Miller et al., 2010) dedicated to the assembly of NGS raw data, we can distinguish two discreet computational approaches; mapping reads to a template genome and *de novo* assembly. Assembly via mapping to a known genome as reference can provide very reliable results for sequencing projects dealing with single-cell samples as it can bypass performance issues originating from sequence repeats, short length of reads, low coverage of sequencing, etc. (Scheibye-Alsing et al., 2009). It is mainly driven by the choice of the reference genome which has to be as phylogenetically related to the sequenced sample as possible. *De novo* assembly is by far the most computationally intensive task (Scheibye-Alsing et al., 2009) as it requires algorithms that perform all possible comparisons between the millions of reads in order to detect any overlaps between them; a method referred to as overlay-layout-consensus (OLC). Although the *de novo* assembly endeavor has been simplified by novel algorithms abandoning the OLC method and exploiting mathematical concepts such as de Bruijn graphs (Zerbino and Birney, 2008; Peng et al., 2011), it still heavily depends on the quality of the sequencing protocol (read length, sequencing depth, etc.). Nevertheless, because of the immense diversity of the genomic content in a metagenomic sample, utilization of a reference genome is ruled out, making thus the computationally intensive task of *de novo* assembly the sole practical alternative, at least at the first steps of an analytical effort, when there is no prior knowledge about the sequences pertaining the sample.

#### Open reading frame/gene detection

The functional patterns, which form the response of all living organisms in an environmental niche as well as their symbiotic or competitive interactions, are encapsulated their genetic code, where all necessary information for functions such as nutrition, chemotaxis, adaptation to hostile environments and proliferation, is encoded in the form of genes. In this sense, the identification of genes within a genome, through apt mapping of each gene to its sequence or sequences, is an indispensable step, for its proper functional annotation and the decipherment of the underlying regulatory mechanisms. Computationally, the detection of genes inside a genome starts with the detection of ORFs, after their evaluation whether they can be translated into functional proteins (so that the respective nucleotide sequences may be considered as candidate gene encoding ones). The algorithms (Yok and Rosen, 2010) that perform this assessment, use various methodologies for gene prediction either from the area of machine-learning (Hoff et al., 2009; Zhu et al., 2010) or not (Noguchi et al., 2008), whereas their underlying operational features, are critically modified according to whether the gene prediction targets prokaryotic or eukaryotic organisms.

#### Gene annotation

Even if all gene sequences of a metagenomic population are distinguished successfully, the abundance of information they contain cannot be exploited without a proper annotation of their function. The most widespread method of annotating a gene sequence is by measuring its homology (Altschul et al., 1990; Kent, 2002) to already known genes taken from public databases (Apweiler et al., 2004; Pruitt et al., 2005; Parasuraman, 2012; Benson et al., 2014). However, as more than 99% of bacterial species cannot be cultured in the lab (Rappe and Giovannoni, 2003; Sharon and Banfield, 2013) and the quantity of metagenomic data that is generated each year continuously expands, these methods are no longer sufficient to predict the function of novel genes. Instead new predictive approaches have emerged, becoming the standard practice for this sort of analysis, such as Hidden Markov Models techniques (Finn et al., 2011) and machine learning methodologies (Tian et al., 2004) that assess sequence similarity, exploiting the whole area of the sequence, seeking profiles (Claudel-Renard et al., 2003) or motives for any known gene with a given functionality, i.e., belong to the same Enzyme Commission (EC) number, rather than prioritizing serial homology.

#### Taxonomic analysis (binning)

**Figure 5:**
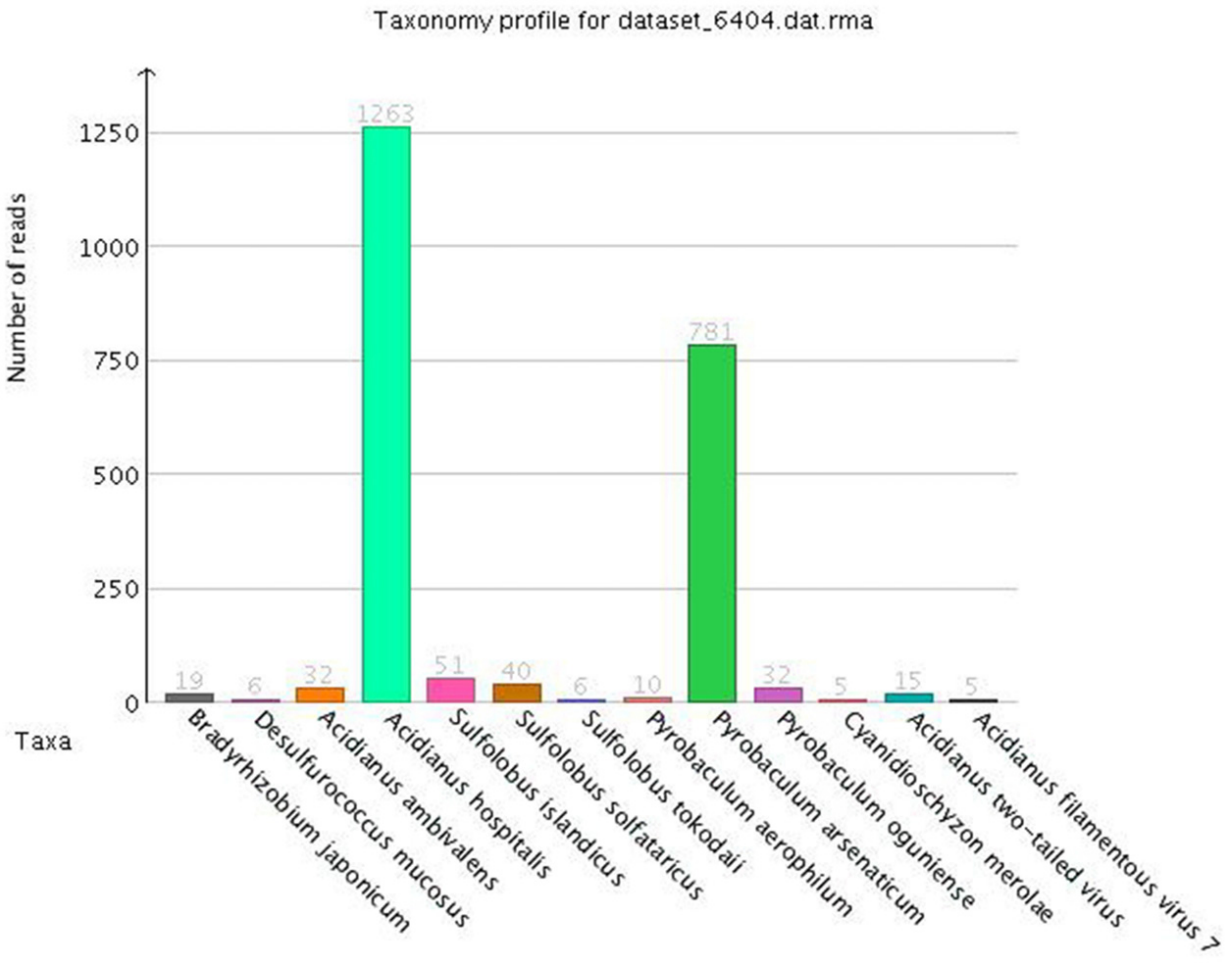
**Taxonomic sorting of sequencing reads from MEGAN software (rank level: “species”)**.

An environmental niche is composed by a broad range of different microorganisms being constantly under evolutionary pressure, which have developed biological interrelations between them, as a means of symbiotic adaptation to the extreme conditions they face. As the DNA extraction from a metagenomic sample gets extracted as a whole, there is no way to separate and segregate beforehand the collected DNA, according to the organism it originated from. Nonetheless this challenge may be addressed computationally, sorting raw sequencing reads taxonomically (Figure 5) and phylogenetically (Weisburg et al., 1991; Retief, 2000; Darling et al., 2014) and thus yield conclusive information about the population of the niche, which can be extended subsequently to the assembled contigs and genes. This process is called taxonomic binning (Droge and Mchardy, 2012) and there are numerous tools (Mohammed et al., 2011; Pati et al., 2011; Luo et al., 2014; Wang et al., 2014) that rely on homology based or composition based approaches (Rosen and Essinger, 2010).

#### Comparative integrative analysis

When different metagenomic datasets are brought together, their overall diversity, which reflects the diversity in the corresponding environmental niches, can be examined computationally. The available tools (Huson et al., 2007; Markowitz et al., 2008; Meyer et al., 2008) for this task incorporate algorithms that compare the functional and taxonomical content of the different datasets and examine if the detected differences are statistically significant.

#### Data management

Following the massive advances of NGS technologies, the generated data from each sequencing analytical job can now reach the order of several gigabytes (GB) or even terabytes (TB) in size(Richter and Sexton, 2009). Moreover if elaborate analytical workflows like the aforementioned are applied, they yield similarly voluminous chunks of processed metadata (in some cases even at a higher order of size e.g., gene annotation). Thus, it is imperative for computational infrastructures, in the form of repositories, to integrate in a single environment, numerous algorithmic workflows that addressing versatile processing tasks together with advanced relational database management functionalities, in order to ensure easy data access, iterative comparative processing and integration of similar information from other datasets. Such infrastructures are now feasible by exploiting the potential of cloud computing (Schatz et al., 2010; Stein, 2010) and provide not only the necessary disk space for large data management but also the appropriate processing capacity for heavy duty bioinformatic tasks.

## Current solutions

Each of the aforementioned tasks not only requests high processing power and storage capacity but also an in depth knowledge of regarding the proper application of computational methodologies from a broad spectrum of fields (information theory, signal processing, systems theory, statistics, programming) along with a yearlong experience in order to produce reliable results. This is why, there is an earnest need for metagenomic analysis platforms introducing automated, workflows for various processing goals, integrating tools in the form of services, operative inside processing pipelines. This has resulted into the development of various pipelines (Almeida et al., 2004; Harrington et al., 2010; Angiuoli et al., 2011) dedicated to the analysis of single organism genomic data. However, the exploitation of NGS technologies in metagenomic analysis has set off the limitations of similar solutions developed for single organism data, for the sake of metagenomic projects. Therefore, for the purposes of this review we will skip the reference to any single-genome tool and will only appraise the most recent pipelines (i.e., frameworks that incorporate two or more tools in consecutive running order) developed for the analysis of metagenomic sequencing datasets. We will also omit pipelines (Schloss et al., 2009; Caporaso et al., 2010) dedicated solely to the analysis of 16s rDNA datasets as these are targeting only phylogenetic studies (Weisburg et al., 1991; Woo et al., 2008), or CAMERA (Seshadri et al., 2007) pipeline as it is no longer supported starting from 1st of July 2014. We also exclude MEGAN (Huson et al., 2007) because despite the fact that it targets metagenomic data, it lacks critical tasks (BLASTX, taxonomic and functional analysis) as part of an automated pipeline.

The current bioinformatic arsenal of pipelines able to take up the challenge of analyzing a metagenomic sequencing dataset comprises the following tools (in alphabetical order): (i) CloVR-metagenomics (Angiuoli et al., 2011), (ii) Galaxy platform (metagenomics pipeline) (Giardine et al., 2005; Kosakovsky Pond et al., 2009), (iii) IMG/M (Markowitz et al., 2008, 2014), (iv) MetAMOS (Treangen et al., 2013), (v) MG-RAST (Aziz et al., 2008; Meyer et al., 2008), (vi) RAMMCAP (Li, 2009), and (vii) SmashCommunity (Arumugam et al., 2010).

### CloVR-metagenomics

CloVR-metagenomics (CloVR: Cloud Virtual Resource) is a desktop application for automated sequence analysis, which requires two different inputs; a set of fasta-formatted files (raw sequencing data), and a tab-delimited metadata file which provides sample-associated information for comparative analysis. Local installation requires a Virtual Machine (VM) player in order to boot the appropriate VM image available by their website. For a cloud-based instance, users can use the Amazon Cloud where they find an available Amazon Machine Image (AMI) from the Request Instances Wizard. The pipeline initiates by clustering redundant sequence reads with UCLUST (Edgar, 2010) and uses BLAST (Altschul et al., 1990) homology searches against COG (Tatusov et al., 2000) and RefSeq (Pruitt et al., 2005) databases for functional and taxonomic annotation respectively. The resulting data from the two different analyses are transferred as input to the integrated Metastats program for detection of differentially abundant features (White et al., 2009). Finally integrated custom scripts in R language (*The R Project for Statistical Computing^2^*) are utilized in order to normalize taxonomic or functional counts for clustering and for visualization purposes. The main advantage of CloVR's setup is that it provides the user with the option of using local resources or to access a cloud provider for additional computational capacity. A potential downside of the platform is the lack of quality control, assembly and gene detection tools (which are available only in the single-genome and 16S-rRNA versions of the software) making it highly dependent on the read length of the sequencing datasets.

### Galaxy platform (metagenomics pipeline)

Galaxy is an open-source, generic framework for the integration of computational tools and databases into a cohesive collaborative workspace, being developed primarily for data intensive biomedical research. A free Galaxy public server (*Galaxy^3^*) is available but a user can download and install an instance on his/her server for exploitation of local resources, tools and databases in order to create custom workflows. Local installation requires only the downloading of the latest release and the initiation of the local instance can be done by running the appropriate BASH (*BASH—The GNU Bourne-Again SHell^4^*) script (run.sh) included in the downloaded directory. A Galaxy workflow for metagenomic datasets was published (Kosakovsky Pond et al., 2009) that requires as input a single dataset of raw sequencing reads and performs an automated series of analyses exploiting specific integrated tools. Those analyses include: (i) quality control and filtering of the reads (custom tool), (ii) text editing and data format converting (custom tools), (iii) homology search against NCBI-nt database (Megablast, Altschul et al., 1990), (iv) taxonomic analysis (custom tools), and (v) visualization of results (custom tools). The biggest advantage of this platform is besides the rich collection of workflows it provides, the capability it offers, via its local installation, to each user to build customized workflows integrating any customized tools of his/her choice (third party or proprietary) that can handle a very wide range of analytical tasks, while simultaneously providing a very friendly user interface. However, in order that a full local installation is achieved, sophisticated, far from trivial, programming expertise rendering the solution inappropriate for other than proficient users. Nevertheless, as the platform becomes more and more popular, many scientific groups develop their own tools and integrate them into new workflows (Pilalis et al., 2012), rendering them available to the relevant communities of users. These workflows provide automated metagenomic analyses that cover from sequence assembly to protein annotation even enzymatic functional classification via machine learning methodologies (Koutsandreas et al., 2013).

### IMG/M

IMG/M is an experimental metagenome data management and analysis system that provides a genome database from bacterial, archaeal and selected eukaryotic organisms and a suite of tools for data exploration and comparative data analysis. The data exploration tools facilitate advanced search queries in assembled sequence data for genes, for the contigs and scaffolds where they originated from as well as their associated functional characterizations (COG, Pfam, Finn et al., 2014, etc.). The comparative data analysis suite contains (i) profile-based selection tools, (ii) gene neighborhood analysis tools, and (iii) multiple sequence alignment tools that can elucidate the gene content and phylogenetic profile of any metagenomic sample. This platform constitutes a very robust and user friendly system for publishing and managing a user's (meta) genome via its web server's graphical user interface (GUI) as well as performing further functional annotation on it, while exploiting their cloud infrastructure. Nevertheless, the burden of quality control of the raw reads as well as the assembly task still befalls on the user. IMG/M is designed for assembled metagenomes only with no supporting tools for the tasks up to assembly. Local installation is not available and all users need to have an IMG Account which can be requested from IMG website.

### MetAMOS

MetAMOS is a metagenomic assembly and analysis pipeline that accepts either raw sequence reads as input or already assembled contigs. Installation requires downloading the latest version and running a Python script (INSTALL.py) included in the release, which automatically handles the whole process. The modules of this pipeline make up a complete analytical workflow that includes: (i) quality control using two different tools (*FASTX-Toolkit^5^*, *Babraham Bioinformatics - FastQC^6^*), (ii) sequence assembly to contigs with eight different assembly methods exploiting four different assembly tools (Zerbino and Birney, 2008; Peng et al., 2011; Treangen et al., 2011; Xie et al., 2014) and to scaffolds with Bambus 2 (Koren et al., 2011), (iii) assembly assessment using a short read aligner tool (Langmead and Salzberg, 2012) and a sequence repeats detection tool(Treangen et al., 2009), (iv) ORF/gene detection with three different available tools (Rho et al., 2010; Zhu et al., 2010; Kelley et al., 2012), (v) gene annotation with seven different available tools (Altschul et al., 1990; Bo et al., 2010; Brady and Salzberg, 2011; Finn et al., 2011; Parks et al., 2011; Darling et al., 2014), and (vi) result visualization using Krona (Ondov et al., 2011). MetAMOS's main strength is the large variety of tools that can be integrated into the workflows, in order to enable a complete automated analysis of any sort of metagenomic dataset, either it constitutes raw sequencing reads or assembled contigs and scaffolds. However, the access to its rich collection of tools is seriously hindered by the lack of a user friendly interface as all tasks must be executed from the linux command line shell, whereas their parameterization requests invocation of appropriate scripts.

### MG-RAST

This pipeline supports both raw sequence reads datasets or already assembled contigs, as input. Local installation is not available as it is offered as an online service for which the user must register in order to upload metagenome datasets and to create jobs. The modules of the automated pipeline comprise four main tasks: (i) normalization of the data, (ii) parallel screening of the sequences against public databases (Maidak et al., 2001; Wuyts et al., 2002; Leplae et al., 2004; Overbeek et al., 2005; Desantis et al., 2006; Meyer et al., 2009), with predetermined default search parameters, for potential protein encoding genes and coding elements, (iii) computation of the resulting data in order to assign functional annotations and taxonomic assignments, and (iv) visualization of results via the integrated SEED Viewer. During the implementation of the pipeline, all job-relevant resulting data are incrementally stored in flat file and SQLite (*SQLite^7^*) format for optimal data management based on relational database technology. The results from the previous steps can be utilized for comparative metagenomic analysis of the original dataset against other metagenomes or complete genomes derived from the SEED environment. What makes this platform attractive to the user is that similar to IMG/M, it provides a user friendly GUI behind a web server that makes the handling of the data and its analysis as intuitive as possible. It also provides numerous tools both for functional analysis and for comparative genomics and it can handle both assembled and not assembled sequence data. The only thing missing from the pipeline are the appropriate modules for raw read quality control and assembly tasks but either than that it constitutes an easy to use and well established functional and taxonomic annotation system that fully exploits the potential of public sequence databases.

### RAMMCAP

RAMMCAP (RAMMCAP: Rapid Analysis of Multiple Metagenomes with a Clustering and Annotation Pipeline) is a metagenomic platform, which workflows enable a complete metagenomic analysis, emphasizing in the programmatic optimization so that the computational cost of the various processing tasks, is minimized. Installation requires downloading the latest version of the package which includes all the essential programs, scripts, and databases. Each of the required programs of the pipeline must then be compiled and installed separately in order to be able to be called upon by the automated pipeline. This pipeline, works with raw read datasets from one or more metagenomic samples, whose sequences are clustered together using CD-HIT (Fu et al., 2012) algorithm. Parallel to clustering the reads, an ORF detection task is implemented, on the raw reads, using a local algorithm (ORF_finder) followed by yet another clustering of the resulting protein sequences. For the clustered and original amino-acid sequences, two parallel workflows are run for similarity detection against Pfam, Tigrfam, (Haft et al., 2001) (HMMER tool) and COG (RPS-BLAST tool) databases generating the subsequent annotation. The final results from (i) clustered raw reads, (ii) database results from clustered protein sequences, and (iii) database results from unclustered protein sequences are examined for statistical comparison of the metagenomes and visualization of their differences. The RAMMCAP pipeline was available as a web service via the CAMERA framework but since the latter has been discontinued it is now only available as a standalone tool for local installation. As is the case with MetAMOS, RAMMCAP's potential gets thwarted by the lack of user friendliness toward the inexperienced user. There is no GUI for the pipeline and its installation and run require a user somewhat more inclined to (bio)informatics. The lack of an integrated assembler also renders it highly dependent to the sequencing read length when it comes to the ORF detection tasks. Besides that it is considered a highly optimized solution in regards to CPU processing and memory demands for comparative metagenomic analysis.

### Smashcommunity

SmashCommunity can be considered as the metagenomic version of its predecessor SmashCell (Harrington et al., 2010), a software designed for the analysis of high-throughput single cell-amplified microbial genomes. Installing SmashCommunity requires the user to download the latest version of the package and to compile/install it using the usual BASH commands (configure, make, make install). Before installing the pipeline the user must also install a list of prerequisite programs and databases that are essential to the various modules of the workflow. This is facilitated by running the BASH scripts (e.g., install_dependencies.ubuntu.sh) included in the release. The required input for this pipeline is raw read datasets from 454 or Sanger sequencing technologies (i.e., long read sequence data). The automated workflow includes integrated tools for: (i) sequence assembly (Myers et al., 2000), (ii) gene detection (Noguchi et al., 2008), (iii) phylogenetic annotation of raw reads (Altschul et al., 1990; Wang et al., 2007; Finn et al., 2011), (iv) functional annotation of detected genes (Altschul et al., 1990; Powell et al., 2014), and (v)comparative analysis (Retief, 2000). Each tool of this workflow is integrated in the automated pipeline via a wrapper script written in Perl^8^ (Stajich et al., 2002) language for facilitating the input/output (I/O) of data between different tasks. SmashCommunity can be considered an “all-inclusive” bioinformatic package but as with similar packages its greatest strength is also its greatest weakness. The numerous prerequisite tools that make up the complete analytical pipeline need to be manually installed beforehand by the user adding to the complexity of the command-line only package. Plus the assembler's restrictions are passed through the rest of the pipeline making its performance optimum only with long read sequencing data (an issue that will soon be obsolete as even Illumina machines are increasing their read length output with each new sequencer release). Despite that, the most advanced user will find that it is a great solution for the conduct of complete and fully automated metagenomic analyses on a local server with dedicated resources.

## Discussion

In order to assess the potential of each metagenomic pipeline we take into account the range of features each pipeline introduces in order to offer an all-inclusive analysis, as well as the level of complexity of its installation. The main features of a full metagenomic analytical workflow should include: (i) sequencing quality control (ii) metagenomic assembly, (iii) ORF/gene detection, (iv) functional annotation, (v) taxonomic analysis, (vi) comparative analysis, and (vii) data management capabilities. From the pipelines we examined, only MetAmos and SmashCommunity included analytical tools for raw sequencing data whereas the rest mainly focused on detecting and annotating putative gene coding regions, as well as providing taxonomic characterization for the generated metagenome. Assessing the complexity of an installation is a fairly subjective matter, yet as “easy” we consider the installation, where the user doesn't have to perform arduous compilation and dependencies' installation tasks, since those usually require a higher level of informatics expertise. For example we consider complex for the inexperienced user, that of RAMMCAP, as it requires a manual installation of each of the integrated tools of the pipeline contrary of the installation of MetAmos, which is handled automatically through the execution of a Python script. The number of features that constitute each of the above-mentioned pipelines are summarized in Table 1.

**Table 1:**
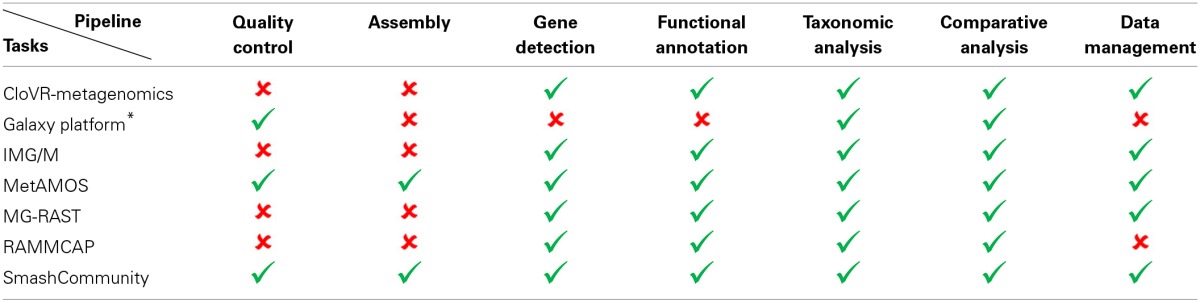
**Display of features of current bioinformatic pipelines for metagenomic data analysis**.

^*^Refers to the metagenomic pipeline of Galaxy.

## Conclusions

The field of Metagenomics holds the promise for the elucidation of the genomic and taxonomic diversity of environmental niches. The rapid advances in sequencing technologies and in the development of algorithms for massive functional annotation of the analyzed genomic content intensify the capabilities of metagenomic analysis, rendering it feasible for an ever-growing number of projects. Powerful, fully automated bioinformatic pipelines lower the entry barrier to the field, through the compilation of numerous workflows, incorporating state-of-the-art algorithms optimized for specific analytical tasks, adjusted also for integration of various datasets, by resolving compatibility issues between them. There are pipelines focusing more on functional and taxonomic analysis, omitting the data-crunchy assembly part while others offer complete solutions where the user simply inputs the data from the sequencer machine and gets a fully annotated genomic report. As expected from other areas of computer science, a trade-off between user-friendliness and efficiency or flexibility of performance is observed here too. The highest the quality and the performance superiority of the workflows, the more profound knowledge they request for their impeccable installation and operation, thus minimizing their accessibility by different scientific communities, short of these skills. On the contrary, pipelines dedicated in resolving smaller, more specific processing tasks, have matured so as to provide very intuitive GUI-based solutions, often via a web server, accessible through the Internet. The broad range of integrative analysis platforms encompasses various pipelines, addressing the pressing need for disparate, versatile, complex, processing tasks. The adopted strategy for the development of efficient workflows, adjustable to varying, yet very specific every time, processing needs, posits on the modularity and transparency of the integrated code, that is the autonomous character of these modules, together with their easiness in integration and user-friendliness in their utilization. Moreover, in order to optimize the computational cost of such processing tasks, parallel processing designs are put forward, aiming to maximally exploit, multi-processor configurations. Among the examined suites of tools (Table 1), we believe, based in our experience for a wide range of metagenomic analysis tasks, that SmashCommunity and MetAmos represent very reliable pipelines, in terms of quality of results, reliability of operation and versatility of tools, for the most experienced users. For those who are analyzing already assembled data for the task of the functional analysis of their metagenome(s), we consider MG-RAST and IMG/M as two very robust and intuitive pipelines. These two aforementioned workflows not only provide tools for a full analysis of any assembled metagenome, but also efficient ways for dissemination of the generated results to the scientific community through a secure database setup.

### Conflict of interest statement

The authors declare that the research was conducted in the absence of any commercial or financial relationships that could be construed as a potential conflict of interest.

## Footnotes

^1^http://www.genome.gov/glossary/index.cfm?id=503 [Accessed].

^2^http://www.r-project.org/ [Accessed].

^3^https://usegalaxy.org/ [Accessed].

^4^http://tiswww.case.edu/php/chet/bash/bashtop.html [Accessed].)

^5^http://hannonlab.cshl.edu/fastx_toolkit/index.html [Accessed].

^6^http://www.bioinformatics.babraham.ac.uk/projects/fastqc/ [Accessed].

^7^http://www.sqlite.org/ [Accessed].

^8^http://www.perl.org/ [Accessed].
